# Genome Sequence of *Salmonella enterica* subsp. *enterica* Strain Durban

**DOI:** 10.1128/genomeA.00399-14

**Published:** 2014-05-08

**Authors:** Daniel A. Russell, Charles A. Bowman, Graham F. Hatfull

**Affiliations:** Department of Biological Sciences, University of Pittsburgh, Pittsburgh, Pennsylvania, USA

## Abstract

We report the genome sequence of *Salmonella enterica* subsp. *enterica* strain Durban, isolated from a patient with salmonellosis and typhoid fever. The strain is closely related to *S. enterica* subsp. *enterica* strain P125109 but differs in loss of the ϕSE20 prophage and acquisition of a prophage similar to ELPhiS.

## GENOME ANNOUNCEMENT

The species *Salmonella enterica* encompasses a large group of pathogenic bacterial strains that includes six subspecies and more than 2,500 serovars (1). *S. enterica* subsp. *enterica* serovar Enteritidis strains are typically associated with food-borne salmonellosis infections and gastroenteritis, and *S. enterica* subsp. *enterica* serovar Typhi strains are associated with typhoid fever. Genome comparisons show that *S. enterica* subsp. *enterica* strains share a common core but differ in their pathogenicity islands, transposons, and prophages, which likely influence their pathogenicity (1). Strains may also carry plasmids that contribute to virulence (2).

While returning home from Durban, South Africa, three microbiologists and a 10-year-old child became sick, exhibiting symptoms that included rigors, high fever (>104°F), vomiting, and diarrhea. Two of the adults and the child received oral ciprofloxacin and regained normal health within several days. For one adult, a phage biologist, the identification of bacteremia with Gram-negative rods prompted hospital admission, after which abnormal liver function was observed and typhoid fever was diagnosed. Following treatment with intravenous fluids and an antibiotic regimen (completed with oral trimethoprim-sulfamethoxazole), the patient was released after 5 days and returned to normal health. Upon discovery of the bacteremia, Gram-negative bacteria were cultured from a blood sample and determined to be *Salmonella* sp. group D. The organism was fully drug sensitive, including to ciprofloxacin and trimethoprim-sulfamethoxazole.

Following propagation and DNA isolation, we determined the genome sequence of strain Durban. Approximately 305 Mb of sequence (~2.3 million reads at ~130-bp average length) from an Ion Torrent PGM were assembled using Newbler 2.6 and the CLC Genomics Workbench. The largest contigs showed high BLASTn similarity to strain P125109, which we used as a scaffold to assist further assembly, resulting in two circular contigs. These contigs were evaluated using AceUtil (C. A. Bowman, D. A. Russell, and G. F. Hatfull, unpublished data) to identify consensus misassignment errors. One contig corresponds to the *Salmonella* genome and is 4,678,926 bp long; the other is a 59,372-bp plasmid (52.5% and 50.0% G+C contents, respectively).

The newly sequenced strain is closely related to the host-promiscuous *S. enterica* subsp. *enterica* serovar Enteritidis strain P125109 (phage type 4 [PT4]; 4,685,848 bp) (3), and the plasmid shares >99% nucleotide identity with *S. enterica* plasmid pSENV (4). We thus designate the new strain *S. enterica* subsp. *enterica* strain Durban. The chromosomes of the two strains show a high level of sequence similarity (>98% nucleotide identity) but differ in two large indels. First, strain Durban lacks the ϕSE20 prophage (40,542 bp) of strain P125109 (3), and second, strain Durban has an additional prophage (34,033 bp) inserted between coordinates 2,755,509 and 2,789,459 of the Durban genome. This prophage is nearly identical (there are two single-nucleotide substitutions) to phage ELPhiS (RE-2010), identified as an active prophage in *S. enterica* serovar Enteritidis strain LK5 (5). The complete genome sequence of strain LK5 has not been reported, so its complete relationship to Durban is unclear. There is no evidence that the professional work of the phage biologist contributed to the acquisition of the ELPhiS prophage by strain Durban.

### Nucleotide sequence accession numbers.

The nucleotide sequence accession numbers are CP007507 and CP007508 for the chromosome and plasmid, respectively.
